# Hierarchy of bond stiffnesses within icosahedral-based gold clusters protected by thiolates

**DOI:** 10.1038/ncomms10414

**Published:** 2016-01-18

**Authors:** Seiji Yamazoe, Shinjiro Takano, Wataru Kurashige, Toshihiko Yokoyama, Kiyofumi Nitta, Yuichi Negishi, Tatsuya Tsukuda

**Affiliations:** 1 Department of Chemistry, School of Science, The University of Tokyo, 7-3-1 Hongo, Bunkyo-ku, Tokyo 113-0033, Japan; 2 Elements Strategy Initiative for Catalysts and Batteries (ESICB), Kyoto University, Katsura, Kyoto 615-8520, Japan; 3 Department of Applied Chemistry, Faculty of Science, Tokyo University of Science, 1-3 Kagurazaka, Shinjuku-ku, Tokyo 162-8601, Japan; 4 Department of Materials Molecular Science, Institute for Molecular Science, Myodaiji, Okazaki, Aichi 444-8585, Japan; 5 Japan Synchrotron Radiation Research Institute, SPring-8, 1-1-1 Koto, Sayo, Hyogo 679-5198, Japan

## Abstract

Unique thermal properties of metal clusters are believed to originate from the hierarchy of the bonding. However, an atomic-level understanding of how the bond stiffnesses are affected by the atomic packing of a metal cluster and the interfacial structure with the surrounding environment has not been attained to date. Here we elucidate the hierarchy in the bond stiffness in thiolate-protected, icosahedral-based gold clusters Au_25_(SC_2_H_4_Ph)_18_, Au_38_(SC_2_H_4_Ph)_24_ and Au_144_(SC_2_H_4_Ph)_60_ by analysing Au L_3_-edge extended X-ray absorption fine structure data. The Au–Au bonds have different stiffnesses depending on their lengths. The long Au–Au bonds, which are more flexible than those in the bulk metal, are located at the icosahedral-based gold core surface. The short Au–Au bonds, which are stiffer than those in the bulk metal, are mainly distributed along the radial direction and form a cyclic structural backbone with the rigid Au–SR oligomers.

## Abstract (web summary)

The hierarchy of bond stiffness may contribute to the thermal properties of metal clusters. Here, the authors use X-ray absorption spectroscopy to study the stiffness of the gold–gold and gold–sulfur bonds in a series of thiolate-protected, icosahedral-based gold clusters.

## Graphical abstract

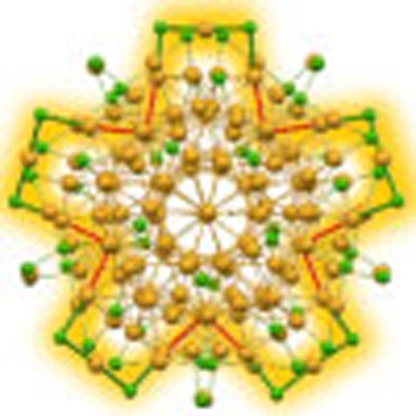

Metal nanoparticles (NPs) exhibit specific thermal properties and phase transition behaviour that are quite different from the corresponding bulk metal^1^,^2^,^3^,^4^. For example, the melting point of a metal NP is significantly depressed with respect to that of the bulk metal^5^. The melting of a metal NP starts with premelting of the surface layer, which then expands towards the inner core and leads to a complete transition to the liquid^6^. These size-specific thermal behaviours are explained in terms of the large surface energy of a metal NP. In other words, a crucial factor that governs thermal behaviours is the hierarchy of the bonding within NPs; metal–metal bonds on the surface are generally softer than those within the core^3^,^7^,^8^. The thermal behaviours of metal NPs are not only affected by their size, but also by the atomic packing of the core^3^,^7^,^8^,^9^,^10^,^11^,^12^,^13^,^14^,^15^,^16^ and the interaction with the environment, such as an organic ligand^17^ or solid support^3^,^18^,^19^. For example, the vibrational spectrum of a metal NP with an icosahedral (Ih) structure has a component with a vibrational frequency that is higher than those of other structures such as cuboctahedra and decahedra, which suggests the formation of stiff bonds within the NPs^7^. The influence of surface adsorbates on the thermal properties of metal NPs has been ascribed to a change in the bond stiffness; Pt–Pt bonds of small Pt NPs are softened by hydrogen adsorption, but stiffened by oxidation^19^ or capping with a polymer^17^. However, an atomic-level understanding of how the bond stiffnesses are affected by a variety of structural parameters has not been attained to date because of the experimental difficulties in defining the atomic packing of a metal NP and the interfacial structure with the surrounding environment.

Recently, a series of thiolate (RS)-protected gold clusters Au_*n*_(SR)_*m*_ with well-defined compositions have gained attention as ideal platforms to study the structure–property correlation and the size-dependent evolution of properties^20^. Among others, Au_*n*_(SR)_*m*_ with (*n*, *m*)=(25, 18), (38, 24) and (144, 60) have been studied most extensively as prototypical systems. Single-crystal X-ray diffraction (XRD) analysis revealed that Au_25_(SR)_18_ and Au_38_(SR)_24_ have icosahedral Au_13_ and bi-icosahedral Au_23_ cores, respectively, which are protected by combinations of –SR–(Au–SR)_2_– and –SR–Au–SR– oligomers (Fig. 1)^21^,^22^,^23^. The atomic structure of Au_144_(SR)_60_ has not yet been determined by X-ray crystallography, but has been theoretically predicted as composed of a hollow icosahedral Au_114_ core protected by 30 –SR–Au–SR– oligomers (Fig. 1)^24^,^25^. In addition, it has been identified that there are distinct Au–Au bonds with different lengths in the range of 2.7–3.3 Å in these clusters (Supplementary Fig. 1). The Au_*n*_(SR)_*m*_ clusters (*n*=25, 38, 144) provide an ideal opportunity to study the hierarchy of the bond stiffness within Au clusters with well-defined atomic structures and surface modification.

In this study, the stiffnesses of the Au–Au and Au–S bonds in Au_*n*_(PET)_*m*_ (phenylethanethiolate (PET)=PhC_2_H_4_S) are examined using Au L_3_-edge extended X-ray absorption fine structure (EXAFS) analysis, which is a powerful tool to study the local structure of the ligand-protected gold clusters^26^,^27^. The temperature dependence of the Debye–Waller (DW) factors of individual bonds are analysed in the framework of the Einstein model, in which a metal cluster is treated as an ensemble of quantum harmonic oscillators with individual Einstein temperatures that depend on the bonding. The hierarchy in the bond stiffness is elucidated. The Au–S bonds are much stiffer than Au–Au bonds in all the clusters and there are two types of Au–Au bonds; one is stiffer and the other is softer than those in the bulk Au. A major portion of the shorter and stiffer Au–Au bonds is distributed within the Ih-based Au core as radial bonds. The stiff Au–Au bonds distributed on the surface of the core are connected by the rigid Au–S oligomers to form a ring structure in all the clusters. These ring structures may act as a rigid framework to enhance the thermal stability of the thiolate-protected Au clusters.

## Results

### Structural analysis

**Figure 1:**
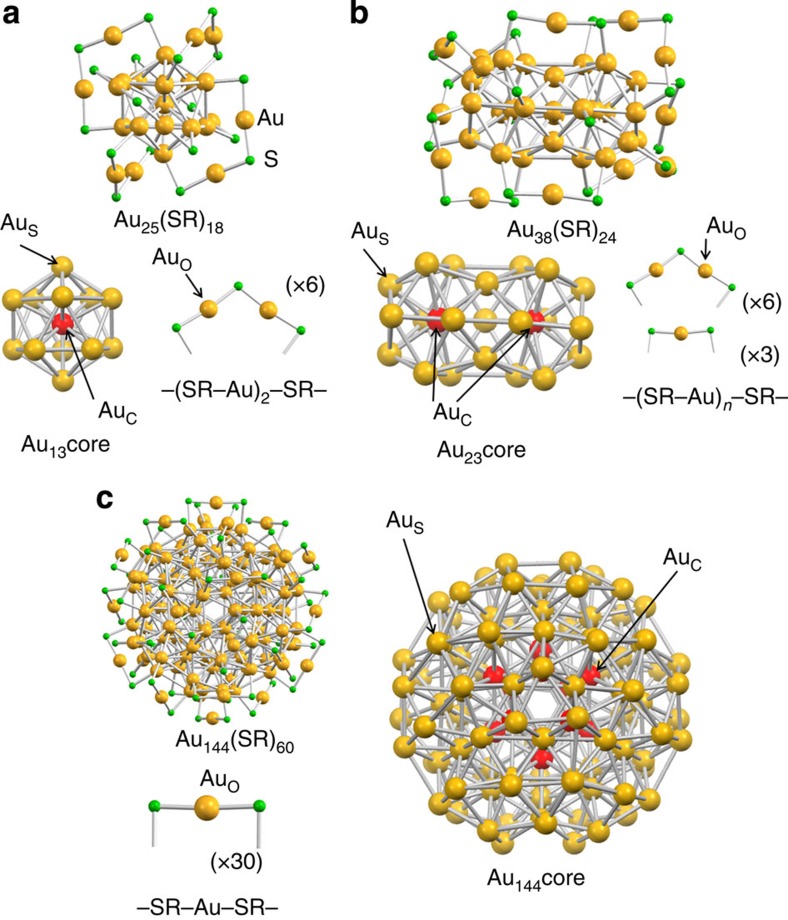
Geometric structures of Au_*n*_(SR)_*m*_ samples. (**a**) Au–S framework of Au_25_(SR)_18_ determined by single crystallography^22^, (**b**) Au–S framework of Au_38_(SR)_24_ determined by single crystallography^23^ and (**c**) Au–S framework of Au_144_(SR)_60_ predicted by density functional theory calculation (ref. 24). Au_C_, Au_S_ and Au_O_ represent Au atoms bonded only to Au atoms, those bonded both to Au and S atoms, and those bridged by two S atoms, respectively for Au_25_(SR)_18_ and Au_38_(SR)_24_. The definition of AuC, Au_S_, and Au_O_ for Au_144_(SR)_60_ is given in the text. *R* is omitted for simplicity.

**Figure 2:**
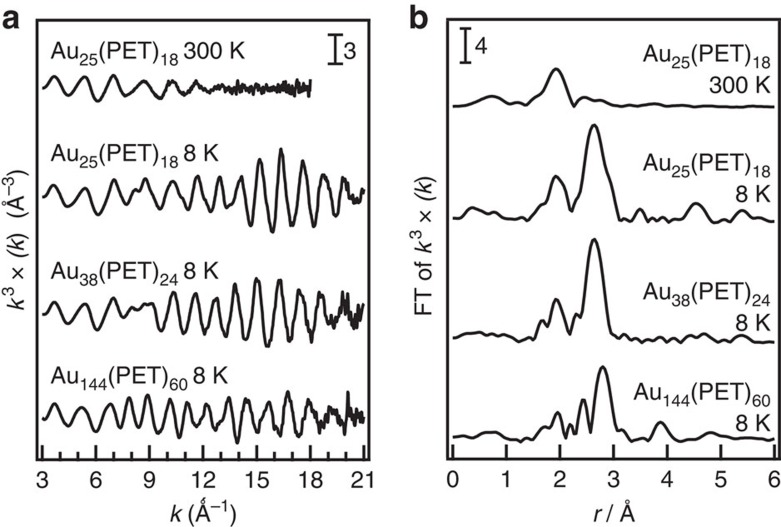
EXAFS oscillations and FT-EXAFS spectra. (**a**) EXAFS oscillations for Au_25_(PET)_18_ measured at 300 and 8 K, Au_38_(PET)_24_ at 8 K, and Au_144_(PET)_60_ at 8 K and (**b**) the corresponding FT-EXAFS spectra.

Figure 2a shows EXAFS oscillations of Au_25_(PET)_18_ measured at 300 and 8 K, respectively. EXAFS oscillation is clearly observed up to the *k* range of 21 Å^−1^ at 8 K, whereas it is damped in the *k* range of >14 Å^−1^ at 300 K. Given that the EXAFS oscillation in the high *k* region mainly originates from heavy atoms, the large amplitude in Fig. 2a suggests that thermal fluctuation of the Au–Au bonds in Au_25_(PET)_18_ is significantly suppressed at 8 K. Figure 2b represents Fourier transformed (FT)-EXAFS spectra of Au_25_(PET)_18_ at 300 and 8 K, respectively. Most notably, the peak due to Au–Au bonds is clearly observed in the bond length (*r*) range of 2.1–3.0 Å at 8 K, in addition to that for Au–S bonds in the *r* range of 1.5–2.0 Å, whereas the Au–Au peak is hardly discernible at 300 K (Fig. 2b). Similar temperature dependence has been reported in the literature^26^ and is attributed to suppression of the thermal fluctuation of Au–Au bonds at low temperature. Previous single crystallographic study revealed that the Au–Au bonds of Au_25_(PET)_18_ are classified into three groups according to their lengths: the Au–Au(*x*) bonds (*x*=1–3) assigned mainly to the Au_C_–Au_S_, Au_S_–Au_S_ and Au_S_–Au_O_ bonds, respectively (see Fig. 1 and Supplementary Note 1; Supplementary Fig. 1a). The average coordination number (CN) and *r* values calculated for the Au–Au(*x*) bonds (*x*=1–3) are summarized in Table 1. Based on this information, curve-fitting analysis of the FT-EXAFS data for Au_25_(PET)_18_ at 8 K was conducted by assuming a single Au–S and three types of Au–Au bonds with different *r* values. However, we found that contribution from the longest Au–Au bonds is significantly smaller than the other two Au bonds due to larger DW factor (see Supplementary Discussion, Supplementary Fig. 2, and Supplementary Table 1). Thus, the FT-EXAFS data was analysed by assuming a single Au–S and two types of Au–Au bonds, Au–Au(S) and Au–Au(L). Their CN and *r* values thus obtained are summarized in Table 1. The CN and *r* values for the Au–S, Au–Au(S) and Au–Au(L) bonds obtained by EXAFS are in good agreement with those of the Au–S, Au–Au(1) and Au–Au(2) bonds determined by single crystal XRD data, respectively. This agreement indicates that low-temperature EXAFS measurements allow quantitative probing of Au–Au bonds within the Au_13_ core: Au_C_–Au_S_ and Au_S_–Au_S_ bonds. The FT-EXAFS spectrum simulated assuming DW factors of 0.0027, 0.0040 and 0.0037 Å^2^ (Supplementary Fig. 3) for Au_C_, Au_S_ and Au_O_, respectively, corresponded well with the experimental spectrum (Supplementary Fig. 4).

Similar temperature dependence was observed in the Au L_3_-edge EXAFS spectrum for Au_38_(PET)_24_ measured at 8 K (Fig. 2a), where a clear oscillation was observed in the *k* range of 3.0–21.0 Å^−1^. The FT-EXAFS spectrum (Fig. 2b) at 8 K has peaks for the Au–S (1.5–2.0 Å) and Au–Au (2.1–3.0 Å) bonds. Single-crystal XRD measurements of Au_38_(PET)_24_ show that 38 Au atoms are categorized into three different sites, Au_C_, Au_S_ and Au_O_, as in the case of Au_25_(PET)_18_ (Fig. 1b). The length distribution of Au–Au bonds obtained from the single crystal structure^23^ is shown in Supplementary Fig. 1b. The Au–Au bonds were classified into three groups with border distances of 2.90 and 3.05 Å: Au–Au(*x*) bonds (*x*=1–3) although there is larger ambiguity in the determination of the border distances than in the case of Au_25_(PET)_18_ (Supplementary Fig. 1a). The Au–Au(*x*) bonds (*x*=1–3) are composed of mainly Au_C_–Au_S_, Au_S_–Au_S_ and Au_S_–Au_O_ bonds, respectively. The average CN and *r* values calculated for the Au–Au(*x*) bonds (*x*=1–3) are summarized in Table 1. The FT-EXAFS spectrum was analysed by assuming a single Au–S and two types of Au–Au bonds with different lengths, Au–Au(S) and Au–Au(L), as in the case of Au_25_(PET)_18_. The CN and *r* values for the Au–S, Au–Au(S) and Au–Au(L) bonds obtained by curve-fitting analysis (Table 1) quantitatively agree with those for the Au–S, Au–Au(1) and Au–Au(2) bonds, respectively. The FT-EXAFS spectrum simulated using DW factors for each of the Au sites shown in Supplementary Fig. 3 reproduced the experimental spectrum (Supplementary Fig. 5).

These analyses demonstrate that low-temperature EXAFS data provides quantitative structural information on the Au bonds within the Au cores of Au_*n*_(SR)_*m*_. Following this successful characterization, we next conducted Au L_3_-edge EXAFS measurement of Au_144_(PET)_60_ at 8 K, of which the structure has not yet been determined by single-crystal XRD. Au L_3_-edge EXAFS oscillation was clearly observed in the *k* range as large as 21.0 Å^−1^, as shown in Fig. 2a. The FT-EXAFS spectrum of Au_144_(PET)_60_ (Fig. 2b) shows a small peak for the Au–S bond in the range of 1.5–2.0 Å and a large peak for the Au–Au bond in the range of 2.1–3.0 Å. The curve-fitting analysis indicates that the data can be reproduced by assuming Au–S (CN=0.9, *r*=2.326 Å), Au–Au(S) (CN=1.2, *r*=2.733 Å) and Au–Au(L) (CN=6.0, *r*=2.870 Å) bonds, as shown in Table 1. The Au–Au bonds are slightly shorter than those of Au_25_(PET)_18_ and Au_38_(PET)_24_, whereas the Au–S bond length is comparable to those of Au_25_(PET)_18_ and Au_38_(PET)_24_. According to density functional theory (DFT) calculations^24^, Au_144_(SCH_3_)_60_ has a hollow icosahedral Au_12_ core. The absence of the central Au atom may be a cause of the reduction in the Au–Au bond lengths compared with those of Au_25_(PET)_18_ and Au_38_(PET)_24_. Supplementary Figure 1c plots the bond lengths between the nearest neighbour Au atoms in Au_144_(SCH_3_)_60_ obtained by DFT calculations^24^. When Au–Au bonds are divided into three groups Au–Au(*x*) (*x*=1–3) with border distances of 2.83 and 3.10 Å, the experimentally obtained CN and *r* values of the Au–S, Au–Au(S) and Au–Au(L) bonds are in quantitative agreement with those of the Au–S, Au–Au(1) and Au–Au(2) bonds, respectively, as shown in Table 1. The EXAFS analysis strongly supports the Au_144_(SCH_3_)_60_ model structure^24^. We then examined which Au–Au bonds contribute mainly to the Au–Au(1) and Au–Au(2) bonds. When the Au atoms of Au_144_(SCH_3_)_60_ are divided into three groups: 12 Au atoms at the hollow icosahedral core (Au_C_), 102 Au atoms located at the middle and the outermost layers (Au_S_) and 30 Au atoms at the oligomer (Au_O_) (Fig. 1), the Au–Au(1) and Au–Au(2) bonds are assigned mainly to the Au_C_–Au_C_ and Au_S_–Au_S_ bonds, respectively. The EXAFS oscillation is again dominated by the Au–Au bonds in the core and the shell, whereas the contribution from the bonds with Au_O_ is negligibly small. The FT-EXAFS spectrum simulated using DW factors for each of the Au sites shown in Supplementary Fig. 3 corresponded well with the experimental spectrum (Supplementary Fig. 6).

### Einstein temperatures for Au–S and Au–Au bonds

**Figure 3:**
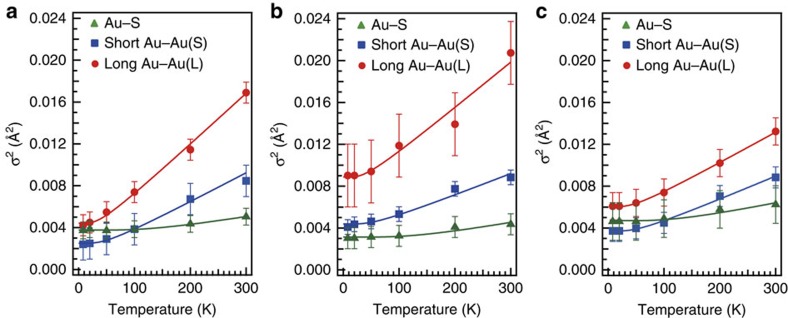
Temperature dependence of the DW factors. Temperature dependence of the DW factors (*σ*^2^) for Au–S, Au–Au(S) and Au–Au(L) bonds. (**a**) Au_25_(PET)_18_, (**b**) Au_38_(PET)_24_ and (**c**) Au_144_(PET)_60_. Solid lines represent the fitting results. Error bars represent the s.d. in the curve-fitting procedure of FT-EXAFS.

The DW factors for the Au–S, Au–Au(L) and Au–Au(S) bonds of Au_*n*_(PET)_*m*_ (*n*=25, 38, 144) were determined by least-squares fit analysis while keeping the CN values the same as those in Table 1, and the results are plotted as a function of temperature in Fig. 3. Details of the analysis are given in Methods section. Figure 3 shows that the DW factors for both the Au–Au(S) and Au–Au(L) bonds increase monotonically with the temperature, whereas those of Au–S bonds remain almost constant in the temperature range of 8–300 K in all clusters. The DW factors for the Au–Au(S) bonds are smaller than those for the Au–Au(L) bonds and are comparable to that for the Au–Au bond in the bulk Au (Supplementary Fig. 7).

The stiffnesses of the Au–S bonds and two types of Au–Au bonds of Au_*n*_(PET)_*m*_ (*n*=25, 38, 144) were evaluated within the framework of the Einstein model. In the framework of the Einstein model, we assume three independent quantum oscillators with different Einstein frequencies (*ω*_E_) for the Au–S, Au–Au(L) and Au–Au(S) bonds. The Einstein temperatures (*θ*_E_) were determined by fitting the temperature dependence of the DW factors for the Au–S, Au–Au(L) and Au–Au(S) bonds. Details of the fitting procedure are given in Methods section. The best-fit results are shown in Fig. 3, and the optimized *θ*_E_ and *σ*_S_^2^ values are listed in Table 1.

## Discussion

In this study, the hierarchy of the bond stiffnesses within Au_*n*_(PET)_*m*_ (*n*=25, 38, 144) was examined using Au L_3_-edge EXAFS. EXAFS results provided quantitative structural information (*r* and CN values) of two types of Au–Au bonds (Au–Au(S) and Au–Au(L)) within the Ih-based Au cores in addition to the Au–S bonds. The temperature dependence of the DW factors of the individual bonds was analysed within the framework of the Einstein model. Einstein temperatures for the two types of Au–Au bonds were determined to be much lower than that of the Au–S bonds in all clusters, which suggests that thiolate-protected Au clusters can be viewed as soft Au cores capped by rigid Au–SR staples. This is in agreement with the results of molecular dynamic (MD) simulation on a model system Au_25_(SH)_18_^−^ that the –SR–(Au–SR)_2_– units are rigid and confine the elastic Au core internally^28^.

**Figure 4:**
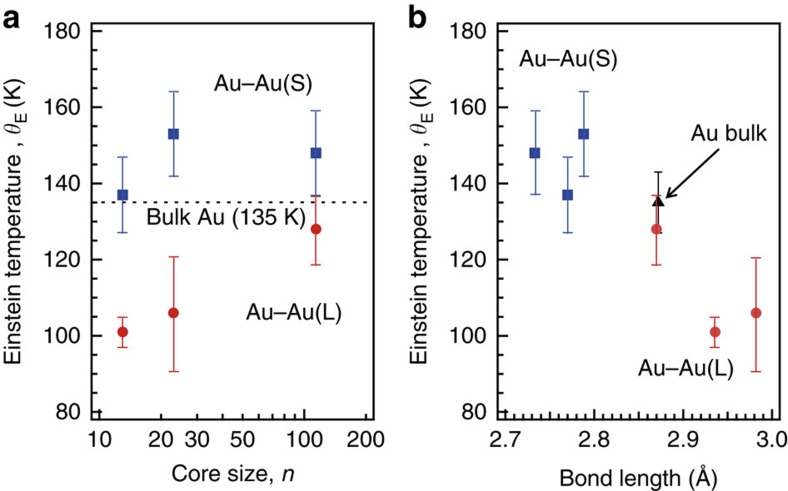
Einstein temperature. (**a**) Einstein temperature (*θ*_E_) as a function of the core size and (**b**) bond length for Au–Au(S) (square) and Au–Au(L) (circle) bonds for core size of *n*=13, 23 and 114. *θ*_E_ and Au–Au bond distance for bulk Au are 135 K and 2.872 Å, respectively. Error bars represent the s.d. in the fitting procedure of *θ*_E_.

More interestingly, two classes of Au–Au bonds with different stiffnesses were identified. Figure 4a shows the Einstein temperatures of the Au–Au(S) and Au–Au(L) bonds as a function of the Au core sizes (13, 23, 114) of Au_*n*_(PET)_*m*_ and that of Au–Au bonds in the bulk Au (135±8 K)^29^. This plot indicates that the Au–Au(S) and Au–Au(L) bonds are respectively stiffer and more flexible than the Au–Au bonds in the face-centred cubic bulk Au, regardless of the cluster size. To the best of our knowledge, this result is the first experimental verification of hierarchy of Au–Au bond stiffnesses in thiolate-protected Au clusters. The correlation suggests that the bond stiffness is related to the bond length. To confirm this hypothesis, Einstein temperatures of the Au–Au bonds were plotted as a function of their lengths in Fig. 4b. The Au–Au bond becomes stiffer with a reduction of the bond length. The Au–Au(S) bonds that are shorter and stiffer than Au–Au bonds in the bulk Au are specific to the Ih core because Au–Au(S) bonds are formed along the radial direction in the icosahedral Au core. The formation of Au–Au bonds in Au_*n*_(PET)_*m*_ that are stiffer than those in the bulk Au is consistent with the theoretical prediction that the vibrational density of states distribution of the Ih cluster has a tail towards a higher frequency than that of bulk Au^4^,^7^,^8^. The Au–Au(L) bonds between the surface Au atoms are more flexible on average than those in the bulk Au, even though they are bonded with the rigid Au–SR oligomers. The above consideration suggests that the Au core of the thiolate-protected Au clusters tends to deform more easily along the lateral direction than in the radial direction. This is consistent with theoretical prediction based on the MD simulation of Au_25_(SH)_18_^−^ that Au core atoms prefer vibrating in the tangential directions as opposed to the radial direction^28^.

**Figure 5:**
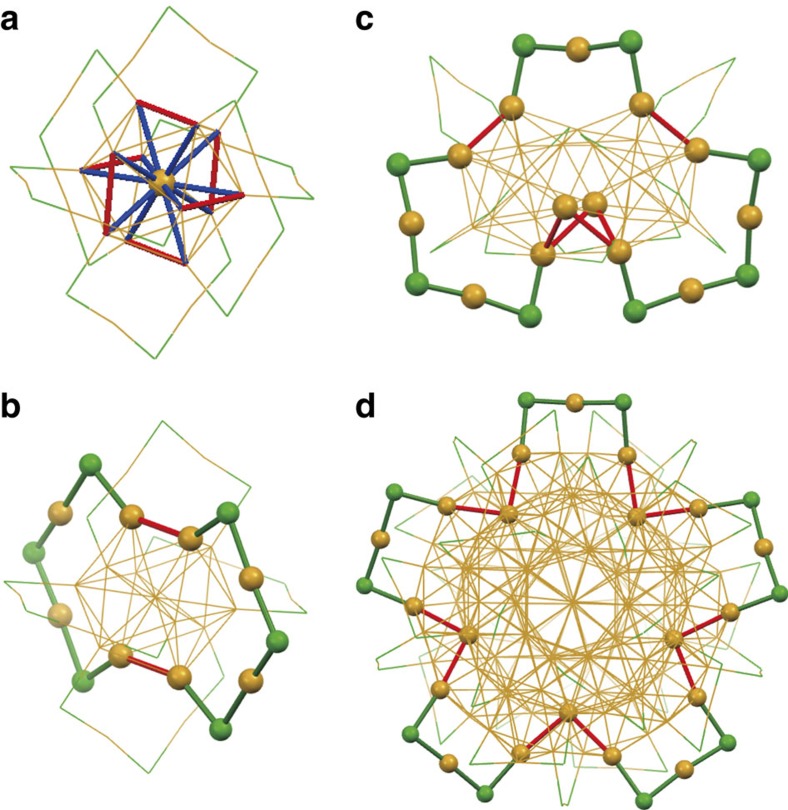
Network structures of stiff bonds. (**a**) Distribution of stiff Au–Au bonds in Au_25_(PET)_18_. Red and blue bonds represent Au_S_–Au_S_ and Au_C_–Au_S_ bonds, respectively. Ring structures in (**b**) Au_25_(PET)_18_, (**c**) Au_38_(PET)_24_ and (**d**) Au_144_(PET)_60_.

Close inspection of the distribution of Au–Au(S) bonds reveals that the stiff Au–Au bonds are distributed not only in the centre of the core, but also on the surface of the core. For example, 6 Au_S_–Au_S_ bonds and 12 Au_C_–Au_S_ bonds in Au_25_(PET)_18_ are categorized as the Au–Au(S) bonds, as shown in Fig. 5a. By bridging these stiff Au–Au surface bonds with the –SR–(Au–SR)_2_– oligomers, a ring structure is formed in Au_25_(PET)_18_ (Fig. 5b). Similar ring structures can be found in Au_38_(PET)_24_ and Au_144_(PET)_60_ (Fig. 5c,d). Tlahuice-Flores *et al*.^30^ also suggested the formation of circular networks (Au_20_Cl_10_) with short Au–Au and Au–Cl bonds as subunits in Au_144_Cl_60_^2+^, which is modelled by replacement of the SR ligands of Au_144_(SR)_60_ with Cl. These results suggest that thiolate-protected Au clusters contain rigid ring subunits that consist of short Au–Au bonds at the surface and Au–SR staples. The formation of these rigid ring structures may act as frameworks to enhance the thermal stability of the thiolate-protected Au clusters.

## Methods

### Chemicals

Hydrogen tetrachloroaurate tetrahydrate (HAuCl_4_·4H_2_O) was purchased from Tanaka Kikinzoku. phenylethanethiol, tetraoctylammonium bromide (TOABr), sodium tetrahydroborate (NaBH_4_), methanol, acetone, dichloromethane and toluene were obtained from Wako Pure Chemical Industries. All chemicals were used without further purification. Deionized water with a resistivity above 18.2 MΩ cm was used.

### Synthesis and characterization

Au_25_(PET)_18_ was synthesized by the methods similar to that in the literature^31^. First, HAuCl_4_·4H_2_O (0.75 mmol) was dissolved in 25 ml tetrahydrofuran solution containing TOABr (0.76 mmol) at room temperature. After stirring for 15 min, phenylethanethiol (4.7 mmol) was added to this solution and the solution was stirred for 15 min. A cold aqueous solution (5.8 ml) containing NaBH_4_ (8.7 mmol) was then rapidly added to the solution and then the solution was stirred at room temperature. After 12 h, tetrahydrofuran solvent was evaporated and the remaining red brown powder was washed with methanol to remove excess thiol and other byproducts. The Au_25_(PET)_18_ cluster was extracted from the dried sample using acetonitrile.

Au_38_(PET)_24_ was synthesized by the methods similar to that in the literature^32^. First, HAuCl_4_·4H_2_O (0.50 mmol) and glutathione (0.25 mmol) were dissolved in acetone (20 ml). This solution was stirred for 15 min at room temperature and for another 15 min at 0 °C. A cold aqueous solution (6.0 ml) containing NaBH_4_ (5.0 mmol) was then rapidly added to this solution and the solution was stirred in ice bath. After 15 min, the acetone was discarded and the residue was dissolved in water (6.0 ml). This solution was then added to the mixture of toluene (2.0 ml) and ethanol (0.30 ml) containing phenylethanethiol (15 mmol). The resulting solution was stirred at 80 °C. After 12 h, the organic solution was evaporated and the resulting black powder was washed by methanol to remove byproducts. The Au_38_(PET)_24_ thus obtained was further purified by the gel permeation chromatography.

Au_144_(PET)_60_ was synthesized by the methods similar to that in the literature^33^. First, HAuCl_4_·4H_2_O (0.60 mmol) and TOABr (0.69 mmol) were dissolved in methanol (30 ml) at room temperature. After stirring for 5 min, phenylethanethiol (3.7 mmol) was added to this solution and the solution was stirred for 15 min. A cold aqueous solution (10 ml) containing NaBH_4_ (6.0 mmol) was then rapidly added to this solution and the solution was stirred at room temperature. After 4 h, the black precipitate was separated by centrifugation and washed repeatedly with methanol or acetonitrile to remove excess thiol, byproducts and the other-sized clusters. The Au_144_(PET)_60_ thus obtained was further purified by gel permeation chromatography.

Chemical compositions and purities of the Au_*n*_(PET)_*m*_ samples were confirmed by a high-resolution Fourier-transform mass spectrometer with an electrospray ionization source (Bruker, Solarix). A 1-mg ml^−1^ dispersion of Au_*n*_(SR)_*m*_ in toluene/acetonitrile (1:1, v-v) was electrosprayed at a flow rate of 800 μl per hour. Ultraviolet-visible-near infrared (UV-Vis-NIR) spectra of Au_*n*_(PET)_*m*_ in toluene were recorded with a spectrometer (Jasco, V-670).

### EXAFS measurements

Au L_3_-edge EXAFS measurements were conducted using the BL01B1 beamline at the SPring-8 facility of the Japan Synchrotron Radiation Research Institute. The incident X-ray beam was monochromatized by a Si(311) double crystal monochromator. A solid sample of each Au_*n*_(PET)_*m*_ cluster was diluted with boron nitride powder, pressed into a pellet, and then mounted on a copper holder attached to a cryostat. All EXAFS spectra were measured in the transmission mode using ionization chambers at 8–300 K. The X-ray energy was calibrated using Cu foil. The EXAFS analysis was conducted using REX2000 Ver. 2.5.9 program (Rigaku Co.) as follows. The *χ* spectra were extracted by subtracting the atomic absorption background using cubic spline interpolation and were normalized to the edge height. The *k*^3^-weighted *χ* spectra within the *k* ranges of 3.0–21.0 Å^−1^ for structural analysis and of 3.0–16.0 Å^−1^ for analysis of temperature dependence of the DW factor were Fourier-transformed into *r* space. The curve-fitting analysis was performed for Au–S and Au–Au bonds over the *r* range of 1.5–3.1 Å. In the curve-fitting analysis, the phase shifts and back-scattering amplitude functions for Au–S and Au–Au were extracted from Au_2_S (ICSD#78718) and Au metal (ICSD#44362), respectively, using the FEFF8 program^34^ by setting *σ*^2^ to 0.0036. This *σ*^2^ value did not significantly affect the phase shift and back-scattering amplitude functions. EXAFS spectra for Au_*n*_(PET)_*m*_ were also simulated using the FEFF8 (ref. 34) programs. Reported crystal structures for Au_25_(PET)_18_ (ref. 22) and Au_38_(PET)_24_ (ref. 23) and the calculated structure for Au_144_(SCH_3_)_60_ (ref. 24) were used for the EXAFS simulations. The theoretical bond lengths for the Au_144_(SCH_3_)_60_ structure were scaled by 0.974 because the Perdew, Burke and Ernzerhof approximation used to the exchange-correlation functional overestimates the Au–Au bond lengths^24^. Different DW factors for Au atoms at different sites, as shown in Supplementary Fig. 3, were used for EXAFS simulations because the DW factors for Au atoms are dependent on the sites shown in Table 1.

### Evaluation of DW factors

The EXAFS equation is generally expressed as:


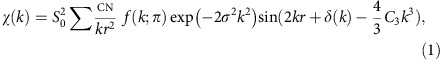


where *S*_0_^2^, CN*, k, r, f*(*k*; *π*), *σ*^2^, *δ*(*k*) and *C*_3_ are passive electron reduction factor, coordination number, photoelectron energy, bond distance, back-scattering amplitude function, DW factor, phase shift, and the third cumulant, respectively^35^. Supplementary Figure 9 shows FT-EXAFS spectra for Au_*n*_(PET)_*m*_ at various temperatures obtained from the temperature-dependent EXAFS data (3.0≤*k*≤16.0 Å^−1^) (Supplementary Fig. 8). It was confirmed by the analysis of bulk Au that the temperature dependence of *C*_3_ must be taken into account in addition to the *σ*^2^ and *r* values (Supplementary Fig. 7)^14^ to reproduce the FT-EXAFS spectra. *C*_3_, *r* and *σ*^2^ values for individual Au–S and Au–Au bonds were determined by least-squares fit analysis while keeping the CN values the same as those in Table 1, and the results are plotted as a function of temperature in Supplementary Fig. 10. In all clusters, the DW factors for both the Au–Au(S) and Au–Au(L) bonds increase monotonically with the temperature, whereas those of Au–S bonds remain almost constant in the temperature range of 8–300 K (Supplementary Fig. 10). The DW factors for the Au–Au(S) bonds are smaller than those for the Au–Au(L) bonds and are comparable to that for the Au–Au bond in the bulk Au (Supplementary Figs 7 and 10).

### Evaluation of Einstein temperatures

The DW factor *σ*^2^ is composed of a dynamic component *σ*_D_^2^ and a static component *σ*_S_^2^, which arises from the thermal atomic oscillation and the temperature-independent structural disorder, respectively:


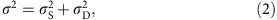


We assume within the framework of the Einstein model three independent quantum oscillators with different Einstein frequencies (*ω*_E_) for the Au–S and Au–Au bonds. The Einstein temperature (*θ*_E_) is defined as:


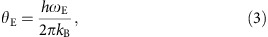


where *h* and *k*_B_ are the Planck and Boltzmann constants, respectively. According to the Einstein model, the dynamic DW factor *σ*_D_^2^ can be fitted using the equation:


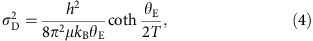


where *μ* and *T* represent the reduced mass of adjacent atoms and the temperature, respectively. The *θ*_E_ values were determined by fitting the temperature dependence of the DW factors for the Au–S, Au–Au(L) and Au–Au(S) bonds using equations (2)–(4), 3, 4. The best-fit results are shown in Fig. 3, and the optimized *θ*_E_ and *σ*_S_^2^ values are listed in Table 1.

## Additional information

**How to cite this article:** Yamazoe, S. *et al*. Hierarchy of bond stiffnesses within icosahedral-based gold clusters protected by thiolates. *Nat. Commun.* 7:10414 doi: 10.1038/ncomms10414 (2016).

## Supplementary Material

Supplementary Information Supplementary Figures 1 - 10, Supplementary Table 1, Supplementary Note 1, Supplementary Discussion and Supplementary References.

## Figures and Tables

**Table 1:** Structural parameters of Au_*n*_(PET)_*m*_ obtained by curve-fitting analysis of Au L_3_-edge FT-EXAFS and single-crystal XRD data.

*n, m*	Method	Bonds	CN*	r (Å)†	σ^2^ (Å^2^)‡	R (%)§	σ_S_^2^ (Å^2^)||	θ_E_ (K)¶
25, 18	EXAFS at 8 K	Au–SAu–Au (S)Au–Au (L)	1.6 (2)1.5 (4)1.5 (6)	2.319 (4)2.770 (3)2.936 (6)	0.0037 (18)0.0027 (11)0.0040 (24)	10.6	0.0017 (3)0.0008 (6)0.0020 (6)	429 (38)137 (10)101 (4)
	Single crystal XRD#	Au–SAu–Au (1)Au–Au (2)Au–Au (3)	1.41.41.92.9	2.332.782.953.16				
38, 24	EXAFS at 8 K	Au–SAu–Au (S)Au–Au (L)	1.2 (2)2.8 (4)1.6 (9)	2.315 (4)2.788 (1)2.982 (15)	0.0030 (10)0.0041 (4)0.0090 (49)	10.7	0.0012 (5)0.0028 (12)0.0066 (5)	416 (57)153 (11)106 (15)
	Single crystal XRD**	Au–SAu–Au (1)Au–Au (2)Au–Au (3)	1.32.71.82.2	2.332.822.983.21				
144, 60	EXAFS at 8 K	Au–SAu–Au (S)Au–Au (L)	0.9 (2)1.2 (5)6.0 (7)	2.326 (8)2.733 (9)2.870 (4)	0.0048 (29)0.0035 (20)0.0059 (13)	12.7	0.0024 (9)0.0021 (5)0.0041 (6)	381 (45)148 (11)128 (9)
	DFT††	Au–SAu–Au (1)Au–Au (2)Au–Au (3)	0.81.55.91.5	2.342.772.923.24				

^*^Coordination number.

^†^Bond length.

^‡^Debye–Waller factor.

^§^*R*=(Σ(*k*^3^*χ*^data^(*k*)–*k*^3^*χ*^fit^(*k*))^2^)^1/2^/(Σ(*k*^3^*χ*^data^(*k*))^2^)^1/2^.

^||^Static component of Debye–Waller factor at 8 K.

^¶^Einstein temperature.

^#^ref. 22.

^**^ref. 23.

^††^ref. 24.
