# Supplementary material for: Hierarchy of bond stiffnesses within icosahedral-based gold clusters protected by thiolates
Source: Nat Commun. 2016 Jan 18;7:10414. doi: 10.1038/ncomms10414 (PMC4735635; doi:10.1038/ncomms10414)
Supplement: Supplementary Information — Supplementary Figures 1 - 10, Supplementary Table 1, Supplementary Note 1, Supplementary Discussion and Supplementary References. [file ncomms10414-s1.pdf]

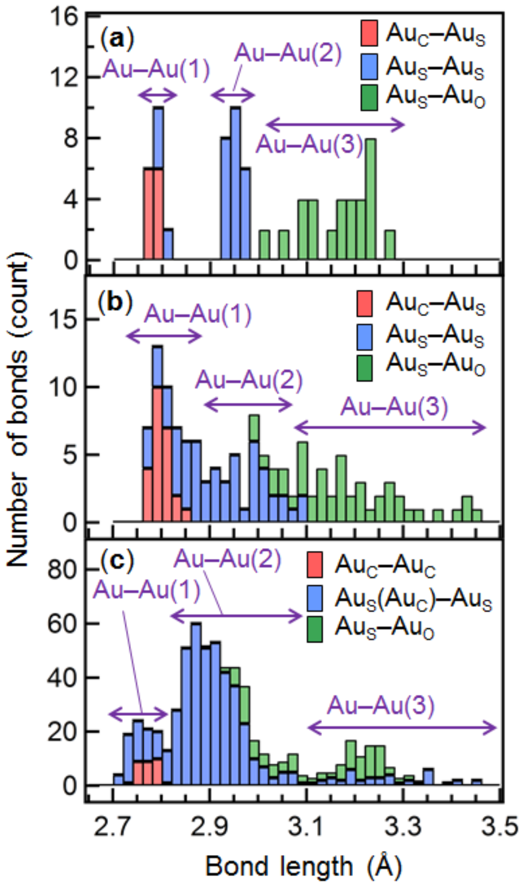

Supplementary Figure 1. Au–Au bond distributions for (**a**) $Au_{25}(PET)_{18}$, (**b**) $Au_{38}(PET)_{24}$, and (**c**) $Au_{144}(SCH_3)_{60}$.

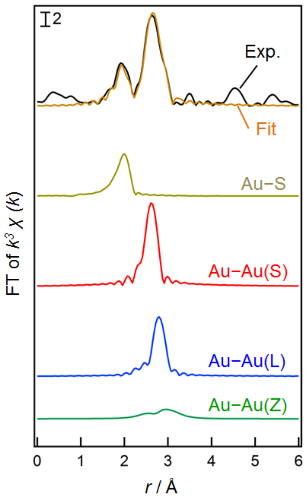

Supplementary Figure 2. FT-EXAFS (Exp.) and fitted (Fit) spectra of $Au_{25}(PET)_{18}$. The fitted FT-EXAFS spectra are composed of four components of Au–S, Au−Au(S), Au−Au(L), and Au−Au(Z).

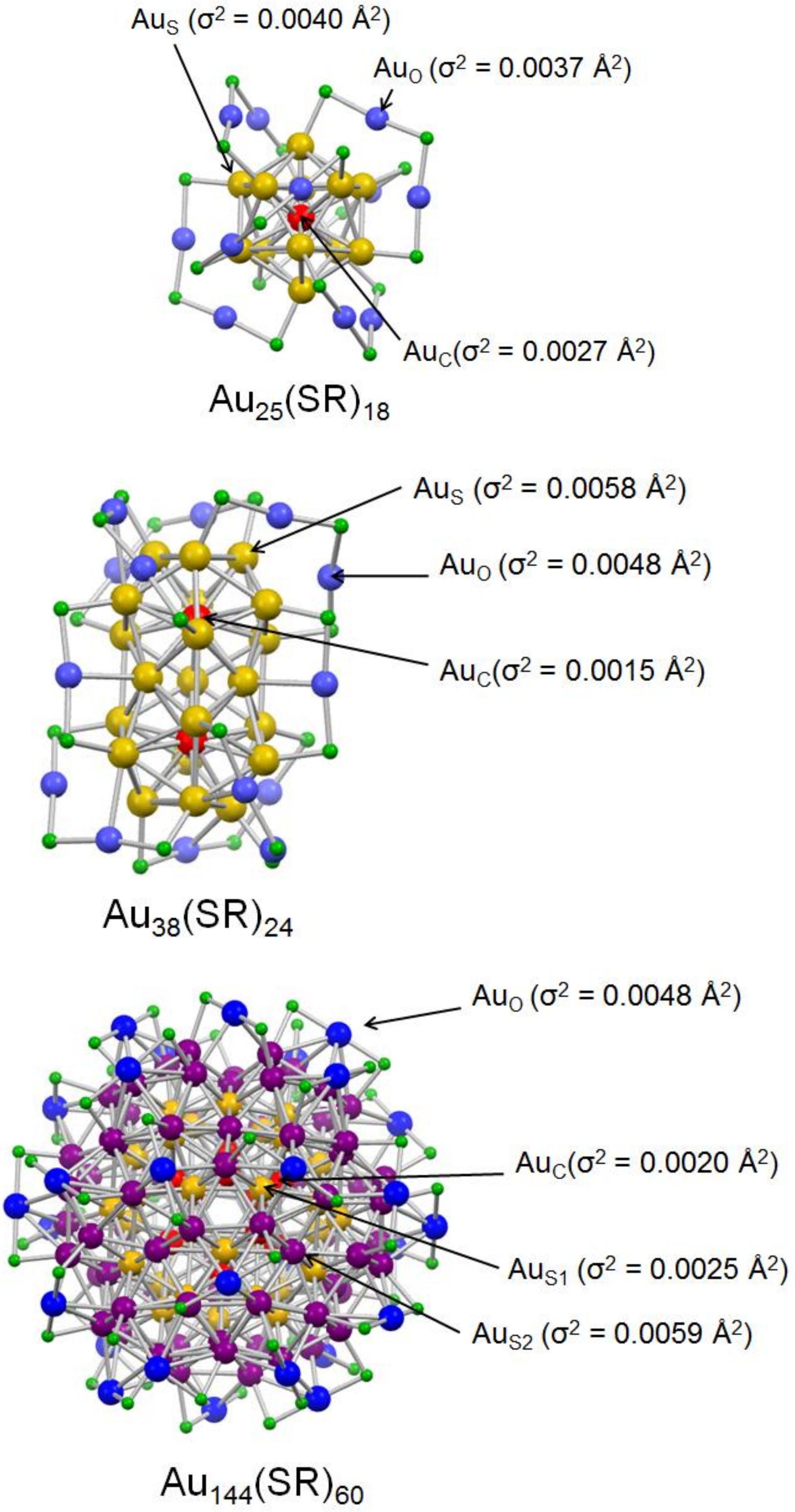

Supplementary Figure 3. Debye-Waller (DW) factors for EXAFS simulations.

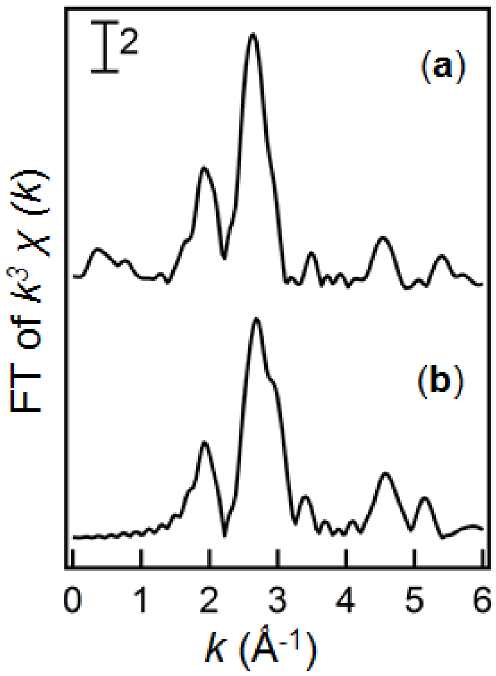

Supplementary Figure 4. Au L_3-edge FT-EXAFS spectra for $Au_{25}(PET)_{18}$ (a) measured at 8 K and (b) simulated using FEFF8.

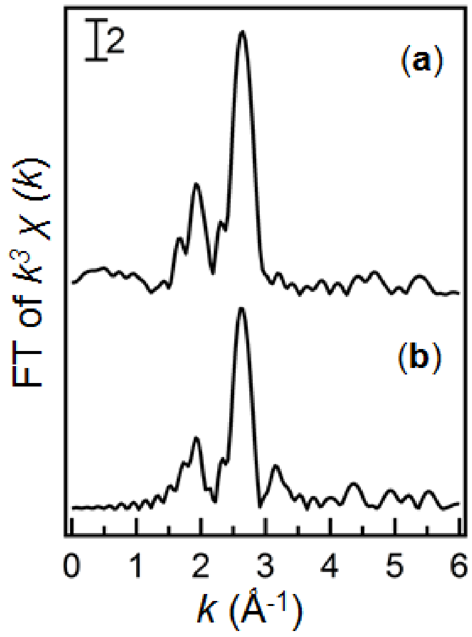

Supplementary Figure 5. Au L_3-edge FT-EXAFS spectra for $Au_{38}(PET)_{24}$ (a) measured at 8 K and (b) simulated using FEFF8.

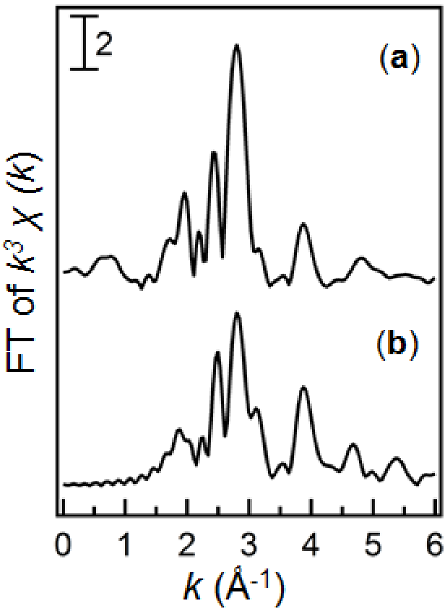

Supplementary Figure 6. Au L_3-edge FT-EXAFS spectra for $Au_{144}(PET)_{60}$ (a) measured at 8 K and (b) simulated using FEFF8.

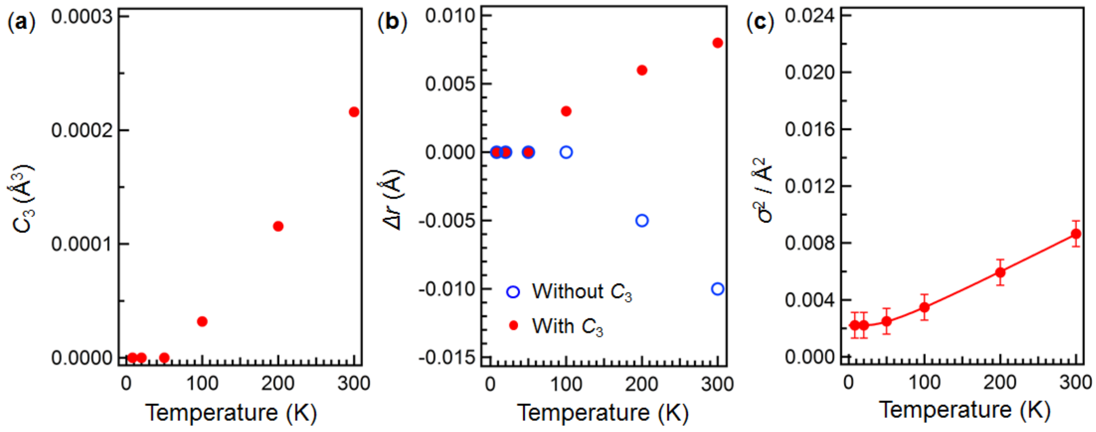

Supplementary Figure 7. Temperature dependence of (**a**) C_3, (**b**) relative Au–Au bond length (Δr), and (**c**) DW factor (σ^2) for bulk Au.

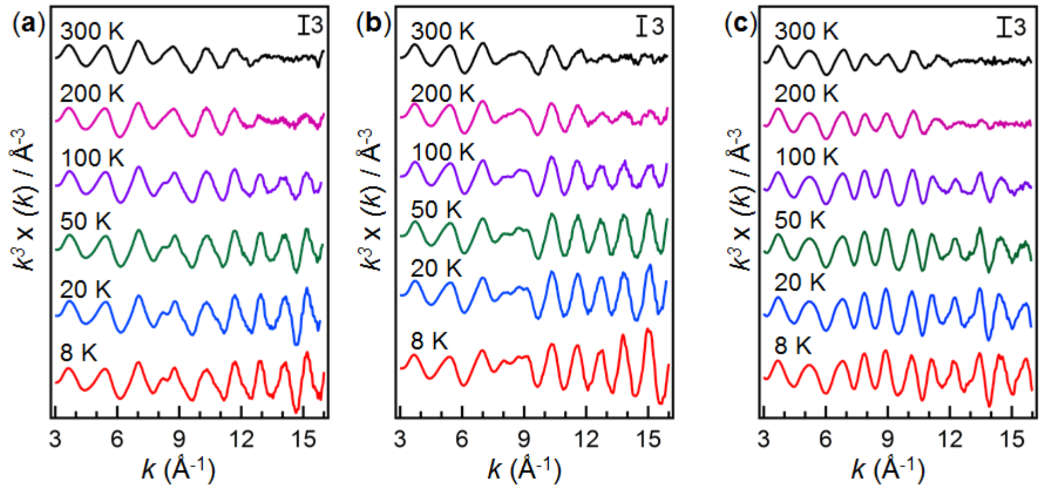

Supplementary Figure 8. Au L_3-edge EXAFS oscillations of (**a**) $Au_{25}(PET)_{18}$, (**b**) $Au_{38}(PET)_{24}$, and (**c**) $Au_{144}(PET)_{60}$ measured at 300, 200, 100, 50, 20, and 8 K.

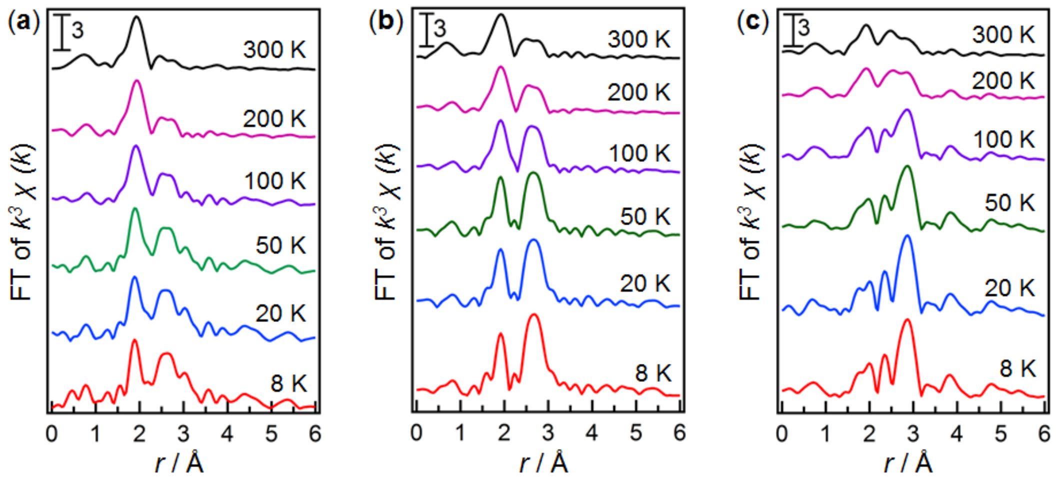

Supplementary Figure 9. Au L_3-edge FT-EXAFS spectra for (**a**) $Au_{25}(PET)_{18}$, (**b**) $Au_{38}(PET)_{24}$, and (**c**) $Au_{144}(PET)_{60}$ at 300, 200, 100, 50, 20, and 8 K.

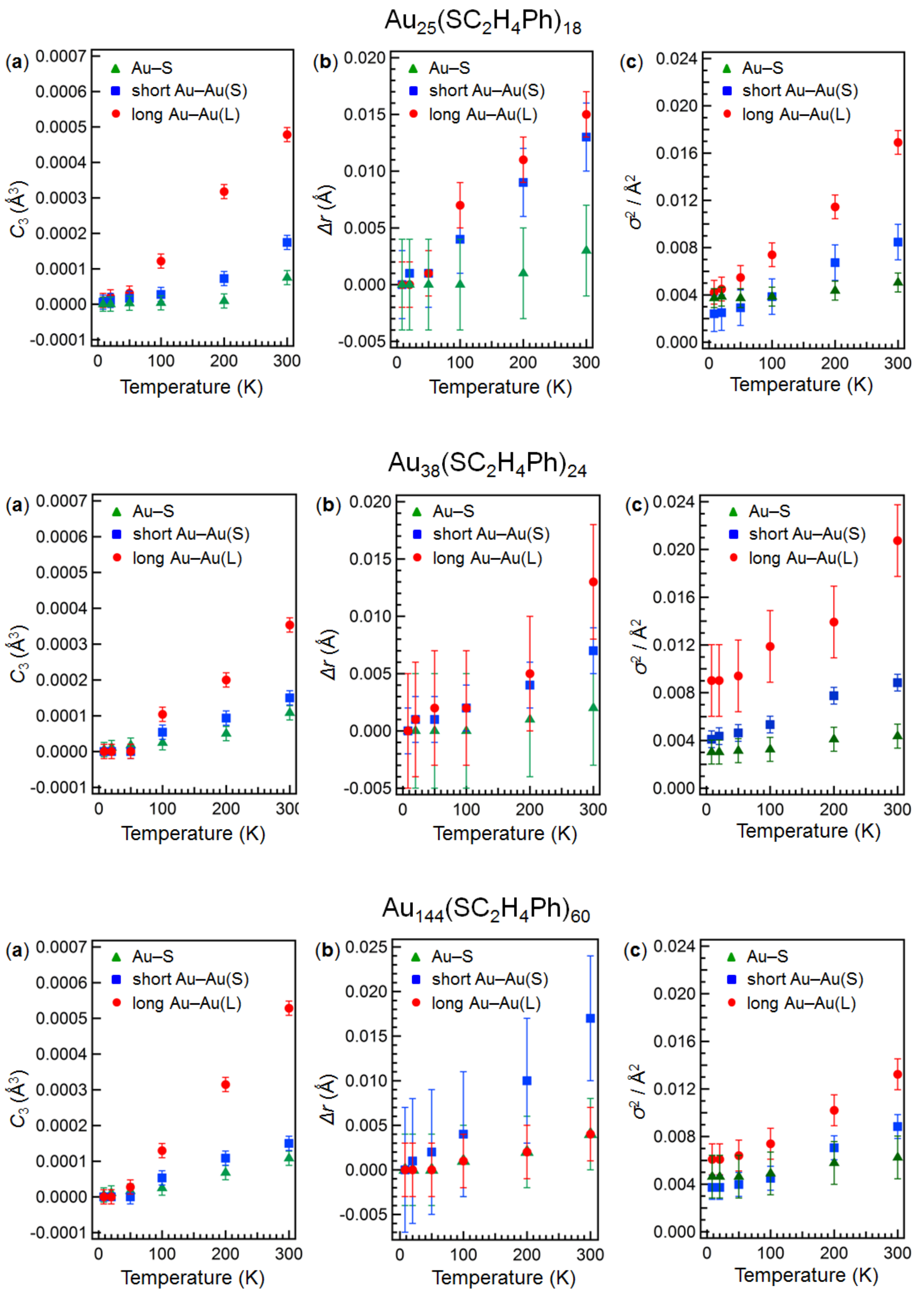

Supplementary Figure 10. Temperature dependence of (**a**) C_3, (**b**) relative Au–Au bond length (Δr), and (**c**) DW factor (σ^2) for $Au_{25}(PET)_{18}$, $Au_{38}(PET)_{24}$, and $Au_{144}(PET)_{60}$.

Supplementary Table 1. Structural parameters of $Au_{25}(PET)_{18}$ obtained by curve fitting analysis of Au L_3-edge FT-EXAFS using a Au−S and three Au−Au bonds.

(*n*, *m*)	Method	Bonds	*CN*[a]	*r* (Å)[b]	σ^2 ($Å^2$)[c]	*R* (%)[d]
(25, 18)	EXAFS at 8 K	Au–S	1.4	2.320(5)	0.0037(18)	14.1
		Au–Au(S)	1.4	2.773(2)	0.0027(11)	
		Au–Au(L)	1.9	2.939(3)	0.0040(24)	
		Au–Au(Z)	2.9	3.124(16)	0.0108(38)	

a) Coordination number. b) Bond length. c) Debye-Waller factor. d) $R = (\Sigma(k^3\chi^{data}(k) - k^3\chi^{fit}(k))^2)^{1/2}/(\Sigma(k^3\chi^{data}(k))^2)^{1/2}$.

Supplementary Note 1: Single crystal structure of $Au_{25}(PET)_{18}$

Single crystal XRD measurements show that there are three Au sites with different local environments in $Au_{25}(PET)_{18}$ (Fig. 1a): (1) an Au atom at the center of the Au_{13} core (Au_C) bonded only to Au atoms, (2) Au atoms at the shell of the Au_{13} core (Au_S) bonded both to Au and S atoms, and (3) Au atoms at the oligomer (Au_O) bridged by two S atoms.[1,2] The bond lengths between nearest neighbor Au atoms determined by single crystal XRD data[2] are plotted as a histogram in Supplementary Fig. 1a. The Au–Au bonds are classified into three groups according to their lengths: 2.75–2.82 (Au–Au(1)), 2.92–2.98 (Au–Au(2)), and 3.00–3.30 Å (Au–Au(3)). The Au–Au(*x*) bonds (x = 1–3) in the histograms can be assigned mainly to the Au_C–Au_S, Au_S–Au_S, and Au_S–Au_O bonds, respectively. The average values of *CN* and *r* calculated for Au–Au(*x*) bonds (x = 1–3) are listed in Table 1.

Supplementary Discussion:

Curve fitting analysis using single Au−S and three Au−Au bonds

We also fitted the FT-EXAFS data of $Au_{25}(PET)_{18}$ using single Au-S and three Au−Au bonds in the range of 1.5−3.3 Å because $Au_{25}(PET)_{18}$ has three types of Au−Au bonds with different bond length (Au−Au(S), Au−Au(L), and Au−Au(Z)). To reduce the number of free-run parameters, we fixed the *CN* values of the three Au−Au bonds to those obtained from the crystal structure (Table 1). The fitting results are shown in Supplementary Table 1 and Supplementary Fig. 10. The bond lengths of Au−S and Au−Au(S), Au−Au(L), and Au−Au(Z) bonds are close to those of Au–S, Au−Au(1), Au−Au(2), and Au−Au(3), respectively, obtained from the single crystal structure. The contribution of Au−Au(Z) bond is much smaller than the others because of its large DW

value. Indeed, the *CN* and DW values for Au−Au(S) and Au−Au(L) fitted by assuming two Au−Au bonds (Table 1) agree well with those listed in Supplementary Table 1.

Supplementary References

1. Heaven, M. W., Dass, A., White, P. S., Holt, K. M. & Murray, R. W. Crystal structure of the gold nanoparticle $[N(C_8H_{17})_4][Au_{25}(SCH_2CH_2Ph)_{18}]$ *J. Am. Chem. Soc.* **130**, 3754-3755 (2008).
2. Zhu, M., Aikens, C. M., Hollander, F. J., Schatz, G. C. & Jin, R. Correlating the crystal structure of a thiol-protected Au_{25} cluster and optical properties *J. Am. Chem. Soc.* **130**, 5883-5885 (2008).
